# Effect of calcium intake and the dietary cation-anion difference during early lactation on the bone mobilization dynamics throughout lactation in dairy cows

**DOI:** 10.1371/journal.pone.0218979

**Published:** 2019-11-27

**Authors:** Pierre Gaignon, Karine Le Grand, Anca-Lucia Laza-Knoerr, Catherine Hurtaud, Anne Boudon

**Affiliations:** 1 PEGASE, Agrocampus Ouest, INRA, Saint-Gilles, France; 2 Phosphea, Dinard, France; 3 CMI, Saint-Malo Cedex, France; National Institute for Agronomic Research, FRANCE

## Abstract

This study investigated the consequences of a low supply of dietary Ca with or without a low dietary cation-anion difference (DCAD) during early lactation on bone mobilization and reconstitution during lactation and on the dynamics of milk Ca content. Fifteen multiparous Holstein cows were distributed among 3 treatments 5 weeks before their expected calving date. These treatments differed based on the provision of diets through the first 10 weeks of lactation. During this period, the control treatment (NCa) consisted of a diet providing 100% of the Ca requirement, with a DCAD of 200 mEq/kg dry matter (DM). The LCa (low Ca) and LCaLD (low Ca, low DCAD) treatments consisted of diets providing 70% of the Ca requirement, with a DCAD of 200 and 0 mEq/kg DM, respectively. After 10 weeks, all cows received the same total mixed ration, which was formulated to meet 100% of the Ca requirement. LCa and LCaLD tended to decrease the body retention of Ca at 3 weeks of lactation compared with NCa but affected neither the dynamics of the blood biomarkers of bone formation and resorption during lactation nor the body retention of Ca at 17 weeks of lactation. Cows almost entirely compensated for the decrease in Ca supply caused by LCa and LCaLD by increasing their apparent digestive absorption of Ca at 3 weeks of lactation, whereas their apparent digestive absorption was unaffected by the treatments at 17 weeks of lactation. Milk production tended to be lower throughout lactation with LCa and LCaLD compared with NCa, with a mean difference of 2 kg/d. The results of this study also indicated that measuring the dynamics of milk Ca content during lactation cannot be considered effective for indirectly estimating the dynamics of bone mobilization in cows. The results also suggested that limited Ca intake at the beginning of lactation may have deleterious effects on milk production.

## Introduction

Dairy cows excrete a large amount of Ca during lactation due to the high Ca content in milk [1], and this Ca flow suddenly and notably increases later during lactation [2]. During the first few months of lactation, the dietary Ca intake is generally lower than the amount of Ca excreted in milk, feces and urine [1], and several responses occur due to this imbalance. The first response is an increase in bone resorption, mediated by the secretion of 2 hormones, namely, parathyroid hormone (PTH) and PTH-related peptide (PTHrP), which allow other organs to use the Ca contained in the mineralized matrix of bone [3]. The net amount of mobilized bone resulting from this increase in bone resorption at the beginning of lactation can reach 10 to 20% of the bone mass during lactation, with the mobilized Ca being replenished later in lactation [4–6]. Other responses, such as an increase in intestinal Ca absorption or a decrease in urinary Ca loss, occur later [7,8], explaining the existence of cycles of bone mobilization and reconstitution during lactation in cows [9–13].

Questions remain about the variations in the amplitude and completeness of these cycles of bone mobilization and reconstitution, their causes, and their consequences for the health and productivity of dairy cows. Incomplete bone reconstitution at the end of lactation can result in a higher susceptibility of cows to the restricted supply of P during the subsequent lactation, resulting in suboptimal production performance, as highlighted for sucker cows by Dixon et al. [6], or possibly higher susceptibility to milk fever at the beginning of the next lactation [14]. Confirmation of both an effect of the amplitude and completeness of the bone cycles on the health and productivity of dairy cows and an effect of the strategy of mineral supplementation on the amplitude and completeness of those cycles would consequently affect the estimated Ca and P requirements of the animals. Current recommendations are based on the principle that daily excretions of Ca and P allow a certain production level to be maintained with minimal fecal and urinary losses and with replacement by an equivalent amount of daily intake of those elements [15–17]. This principle does not consider bone mobilization during early lactation and reconstitution during late lactation that could constitute either an extra supply or a specific requirement, which may facilitate devising supplementation strategies at the scale of the whole lactation-gestation cycle by taking bone reserves into account.

The effects of dietary Ca and P contents and Ca and P supplementation strategies on the amplitude of the bone formation-resorption cycle have been quantified in several experiments in lactating ruminants [10–13,18,19]. The results of a radioisotope study of ewes by Braithwaite (18) suggested that lowering the P and Ca supplies at the beginning of lactation does not affect the bone mobilization amplitude, likely because it is regulated by homeorhesis, but partially impairs bone reconstitution at the end of lactation. However, by employing blood biomarkers of bone mobilization and resorption, it was subsequently established that the dietary P supply had to fall below 70% of the cow’s requirement to induce an increase in bone mobilization that was also accompanied by a dramatic loss of ingestion [10,13,20]. Although the effects of a low Ca supply on bone mobilization and resorption in lactating dairy cows have been reviewed [2,7], only one study quantified such effects throughout the entire lactation period in dairy cows [11]. Through measurements of both retained Ca and P at the animal scale and blood biomarkers, it was shown that both bone mobilization at the beginning of lactation and bone reconstitution at the end of lactation could be limited proportionally to the dietary Ca supply. This finding highlights the need to further quantify how the amplitude of bone mobilization at the beginning of lactation can be amplified in cows with a good level of production and the consequence that it might have for bone reconstitution during the second part of lactation. On the other hand, lowering the dietary anion-cation difference (DCAD) to a value between 0 and -150 mEq/kg dry matter (DM) is a known means of increasing prepartum bone mobilization [21–23] that can also be employed at the beginning of lactation to artificially increase bone mobilization. The optimal DCAD for lactating cows is approximately 200–250 mEq/kg [24].

This lack of studies on the effects of dietary Ca content on bone mobilization and reconstitution throughout lactation in dairy cows could be related to a lack of fast and cost-efficient methods for evaluating the amplitude and eventually the completeness of the cycles of bone mobilization and reconstitution during lactation and gestation in a significant number of cows. Measurements of retained Ca and P at the animal scale [11] and bone biopsies [9] cannot be performed on a significant number of animals. The concomitant use of blood biomarker analyses of bone formation and resorption has increased over the last 20 years, and this interesting method enables monitoring of the relative dynamics of bone formation and resorption during lactation [13,25,26], but its use is limited by the necessity of conducting several blood samplings during lactation with relatively expensive analyses. VanHouten et al. [27] showed that a decrease in Ca intake in mice induced lower Ca secretion in milk and higher bone resorption mediated by PTHrP secretion, with both being mediated by the Ca-sensing receptor (CaSR) in the mammary gland. These results suggest that the monitoring of milk Ca content during lactation could be an inexpensive means of indirectly estimating the dynamics of bone resorption. Data collected during several stages of lactation in dairy cows with different parities [28] suggested that milk Ca and P contents were related to the plasma concentrations of biomarkers of bone formation and resorption, but this possibility requires confirmation.

The objective of this experiment was to quantify the effect of a limited dietary supply of Ca (70% of the French recommendation [15]) in addition to a low DCAD on the amplitude and duration of bone mobilization at the beginning of lactation in high-producing cows. Another objective was to evaluate the consequences for the dynamics of milk Ca and P contents. Our hypothesis was that the restricted Ca supply would greatly increase the amplitude and duration of bone mobilization during early lactation and that the addition of a low DCAD would enhance this increase, which could be an interesting model with which to further evaluate the consequences of increased bone mobilization at the beginning of lactation on subsequent bone reconstitution. Another hypothesis was a decrease in the milk Ca content during this period, concomitant with bone mobilization.

## Materials and methods

### Animals and experimental design

The 3 compared treatments consisted of 3 mineral supplements fed between 5 days after calving and 10 weeks after the beginning of lactation. The Ca content of the supplement was calculated either to fully meet the Ca requirements of the cows according to the INRA feeding system [15], with a DCAD of 200 mEq/kg DM (normal Ca, NCa), or to provide only 70% of the Ca requirements of the cows, with a DCAD of either 200 (low Ca, LCa) or 0 (low Ca and low DCAD, LCaLD) mEq/kg DM. These 3 treatments were compared among the 15 lactating cows according to a completely randomized block design, with lactation stage serving as the blocking factor. Five weeks before the calving date of the cow that was expected to calve first, 18 multiparous Holstein cows were assigned to three groups of 6 cows according to their expected dates of calving. Primiparous cows were excluded from the experiment, and special care was applied to balance the average parity among treatments because parity in dairy cows is a strong determinant of bone formation and resorption throughout lactation [11,28,29] and digestive absorption of Ca [30]. Cows were assigned to the three treatments to allow homogeneous representation of groups and parity within each treatment and to have similar averages of mature equivalent milk productions and milk protein contents between groups, based on levels observed in the first 32 weeks of the previous lactation. The mature equivalent milk production was estimated as equivalent to milk production for a third lactating cow, i.e., 120% of the milk production for primiparous cows and 104% for cows lactating for a second time, as established from the data used by Gaignon et al. [31]. Measurements started 3 weeks before the average expected date of calving for each group and were performed on 5 cows of the initial 6, with the extra cow being kept only for blood analyses to replace a cow whose actual calving date might occur too far from the expected date. The average parity ranks of the cows assigned to the 3 treatments were 3.0 ± 0.46 for NCa, 3.0 ± 0.41 for LCa and 2.4 ± 0.41 for LCaLD, taking into account the current gestation. Their ages at calving were 49.5 ± 5.86 months for NCa, 50.5 ± 5.27 months for LCa and 43.8 ± 5.27 months for LCaLD.

The experiment was conducted at the INRA experimental farm of Méjusseaume (longitude: -1.71°, latitude: +48.11°, Brittany, France) from September 1^st^, 2016, to June 30^th^, 2017. During the experiment, the cows were housed in a free-stall barn cubicle covered with rubber carpet, except during 3 periods of 3 weeks for measurements of Ca retention, during which they were transferred to individual tie stalls (1.4 × 2.0 m). The individual stalls were also covered with a rubber mat and supplied with individual troughs and individual water bowls. During lactation, a total mixed ration (TMR) was distributed twice a day in 2 equal-sized meals at 0830 h and 1630 h. The TMR was distributed by an automatic dispenser into the individual troughs specific to each animal in the free-stall barn and directly by animal technicians in the individual tie stalls. Cows always had free access to the trough and to water during the day. Lactating cows were fed *ad libitum* and offered quantities calculated to allow 10% orts. Orts were weighed daily before the morning feeding. Cows were milked twice a day, at 0630 h and 1630 h. Milk production and DM intake were recorded daily for each individual. Milk composition (fat and protein contents) and somatic cell count were measured twice a week at the evening and morning milkings. Before calving, each cow was fed a fixed amount of diet, which was distributed once per day. Procedures related to the care and use of animals for the experiment were approved by the animal care committee of the French Ministry of Agriculture in accordance with French regulations (project number 7096–20 16082515505689v2).

### Diets

During the experiment, cows were fed 4 or 5 successive diets according to their physiological stage and the treatment to which they were assigned (Table 1). During the dry period, the offered diets were formulated to cover the requirements for net energy of lactation, protein digestible in the intestine and minerals and vitamins of cows according to the INRA recommendations [15], with restricted quantities being offered. The diets offered during the far-off period, i.e., more than 3 weeks before the expected calving date, and the close-up period, i.e., less than 3 weeks before the expected calving date, differed, with the specific objective of lowering the dietary Ca and the DCAD. For the first 5 days of lactation, all cows received the TMR corresponding to the NCa treatment, whose composition was formulated to cover the requirements for net energy of lactation, protein digestible in the intestine, macrominerals, trace minerals, and vitamins of cows according to the INRA recommendations [15], with a target DCAD of 200 mEq/kg DM. The DCAD is defined as the sum of the dietary Na^+^ and K^+^ contents minus the sum of the dietary Cl^-^ and S^2-^ contents, which are expressed in mEq/kg of DM. Five days after calving and until the end of the 10^th^ week of lactation, cows were assigned to one of 3 TMRs corresponding to the compared treatments. The LCa and LCaLD TMRs were formulated to meet the requirements of cows for NE_L_, PDI, and all macrominerals and trace minerals, except for Ca, according to the INRA recommendations [15]. The treatment diets were isocaloric and isonitrogenous, with similar P contents. Differences in the Ca content and DCAD among the 3 TMRs were achieved by formulating different mineral supplements, with all cows receiving the same base ration.

**Table 1:** Dietary centesimal composition and nutritional value.

	From -5 to -3 weeks	From -3 weeks to calving	From 5 to 70 days of lactation			After 70 days of lactation
			NCa	LCa	LCaLD	
Offered amount (kg DM/day)	12	15	TMR *ad libitum*	TMR *ad libitum*	TMR *ad libitum*	TMR *ad libitum*
Ingredients, % of DM						
**Corn silage**	53.7	80.5	70.0	70.2	70.1	72.1
**Hay**	14.6	0.0	0.0	0.0	0.0	0.0
**Wilted and wrapped hay (baleage)**	24.5	0.0	0.0	0.0	0.0	0.0
**Energy concentrate**	0.0	4.1	15.6	15.7	15.1	11.1
**Soybean meal (formaldehyde-treated)**	0.0	0.0	10.3	10.3	10.2	0.0
**Soybean meal**	0.0	10.1	0.0	0.0	0.0	13.1
**Rapeseed meal**	5.9	0.0	0.0	0.0	0.0	0.0
**Straw**	0.0	3.3	0.0	0.0	0.0	0.0
**Urea**	0.0	0.0	1.3	1.3	1.3	0.7
**Mineral feed**	0	0.7	2.9	2.3	3.3	2.5
**Commercial mineral feed**	1.3^1^	1.3^2^				
Mineral feed, g/kg of DM						
**Calcium carbonate**	0.0	0.0	10.7	4.7	4.4	9.9
**Dicalcium phosphate**	0.0	0.0	5.3	4.6	4.4	4.7
**Sodium sulfate**	0.0	0.0	2.7	2.3	2.3	2.4
**Sodium bicarbonate**	0.0	0.0	2.2	2.8	0.0	2.4
**Sodium carbonate**	0.0	0.0	3.1	3.7	0.0	0.0
**Magnesium oxide**	0.0	0.0	0.9	0.9	0.0	1.4
**Hexahydrate magnesium chloride**	0.0	6.7	3.6	3.7	21.5	3.8
**Premix**^**3**^	0.0	0.0	0.2	0.2	0.2	0.2
Nutrient content						
**CP (g/100 g DM)**	11.1	10.5	15.5	15.5	15.5	12.7
**NE**_**L**_ **(MJ/kg)**	3.9	6.4	6.7	6.7	6.7	6.6
**PDI/NE**_**L**_ **(g/MJ)**	9.5	11.0	15.8	15.8	15.8	11.9
**%Ca (g/kg DM)**	0.74	0.42	0.83	0.60	0.58	0.78
**%¨P (g/kg DM)**	0.37	0.30	0.41	0.39	0.39	0.40
**DCAD (mEq/kg DM)**	156.8	34.8	218.0	279.5	0.0	219.9

^1^ Kéomine Repro (Cooperl Hunaudaye, Lamballe, France): 55.7% calcium carbonate, 18.4% monocalcium phosphate, 10.0% magnesium phosphate, 9% cane molasses, 2.4% magnesium oxide, and 4.5% trace elements and vitamins.

^2^ Kéomine Prépa (Cooperl Hunaudaye, Lamballe, France).

^3^ for trace elements and vitamins.

### Blood and milk sampling

For each group, blood was sampled 3 weeks before the average expected date of calving in the group and at 1, 3, 8, 12, 17, 22, 27 and 31 weeks of lactation (average stage of lactation of the group). After being milked, before being fed, and while restrained in self-locking head gates at the feedline, the cows were sampled for blood by venipuncture of the tail vessels. The samples were collected in vacutainer tubes (Monovette, Sarstedt, Nümbrecht, Germany) coated with lithium heparin for Ca and inorganic P (Pi) analyses and in tubes coated with EDTA for osteocalcin (OC) and carboxy-terminal crosslinking telopeptide of type I collagen (CTX) analyses. OC and CTX are biomarkers of bone formation and resorption, respectively [32]. Plasma was recovered after centrifugation at 3,000 *x* g for 12 min within 30 min of sampling and stored at -80°C for OC analysis and at -20°C for the other analyses. Milk samples were collected during the morning milking from total milk before blood sampling. The samples were stored at 4°C for analyses of fat and protein contents and for separation of the N, crude protein, soluble and nonsoluble Ca and P fractions (i.e., nonprotein nitrogen (NPN), noncasein nitrogen (NCN), urea, soluble Ca and P) and frozen at −20°C for analysis of the total Ca and P contents. Additional samples of milk were also collected at the morning and evening milkings and twice per week for determination of milk fat and protein contents (stored at 4°C before analyses), and at weeks 1, 3, 6, 8, 10, 12, 14, 17, 19, 22, 24, 27, 29 and 31 of lactation, samples were collected for analyses of the milk total Ca and P contents (frozen at -20°C before analyses).

### Measurement of Ca and P retention in cows

For each group, all input (feed and water intake) and output (excretion in milk, urine and feces) flows of Ca and P were measured 3 times during the experiment, i.e., 3 weeks before the average expected calving date of the group and 3 and 17 weeks after the average calving date. For each measurement, cows were moved from the free-stall barn 2 weeks before beginning the measurements and sent to individual tie stalls for habituation. The individual tie stalls were located in the same building as the free-stall barn. All cows were kept in the same room and were able to smell and hear each other. The feeding modalities remained similar to those applied in the free-stall barn. To determine the fecal excretion of Ca and P, large trays were positioned behind the cows on day 15 after the entry of the cows into the stall at 0900 h. Gross fecal output was weighed and sampled from day 15 at 0900 h to day 19 at 0900 h. Two representative samples (500 g of fresh feces each) were dried in a forced-air oven (80°C, 72 h) to determine the daily amount of fecal DM excreted. These dried samples were pooled by cow and period for Ca and P determination. The daily volume of excreted urine was measured from day 15 at 0900 h to day 19 at 0900 h by equipping cows with urinary catheters connected by a Tygon tube to a 25-L container, which was closed with a rubber plug. To prevent urine deterioration, 250 mL of sulfuric acid (20% vol/vol) was added to the container. The urine was weighed and emptied daily at 0900 h. Each day and for each cow, a sample of 1% of the daily excreted volume was stored at −20°C. At the end of the experiment, these samples were pooled by animal and by period for further Ca and P content analyses.

### Chemical analysis

Samples of the offered diets, refusals, and feces were ground with a 3-blade knife mill through a 0.8-mm screen. Ash was determined by calcination at 550°C for 5 h in a muffle furnace. Nitrogen concentration was determined by the Dumas method according to the Association Française de Normalisation (AFNOR, 1997) on a LECO apparatus (LECO, St. Joseph, MI). The dietary, fecal, urine and milk Ca contents were measured by atomic absorption spectrophotometry (Spectra-AA20 Varian, Les Ulis, France) with the use of lanthanum chloride solution to dilute the sample and after calcination of the solid samples (500°C for 12 h). Phosphorus contents were determined using a KONE PRO multiparameter analyzer (Thermo Fisher Scientific, Illkirch, France) by the Allen method for P [33]. Milk fat and protein contents were determined by a commercial laboratory using midinfrared analysis (Mylab, Châteaugiron, France). The milk total N (Kjeldahl), nonprotein N (precipitation at a pH of 4.6 with trichloroacetic acid and filtration), NCN (precipitation at a pH of 4.6 with 10% acetic acid and 1 M sodium acetate), and urea (colorimetric analysis) contents were determined according to the methods described in Hurtaud et al. [34]. Plasma OC and CTX concentrations were determined by ELISA with a CrossLaps kit from IDS (Paris, France) for CTX and a kit from Quidel (San Diego, CA) for OC (interassay variation: 3.7%, intra-assay variation: 3.3% for CTX; interassay variation: 5.42%, intra-assay variation: 7.35% for OC).

### Statistical analysis

Data were analyzed using PROC GLIMMIX in SAS [35], with a generalized linear mixed model with repeated values, as described below:

$$Y_{ijk}=\mu +{Treatment}_{i}+{Stage\;of\;lactation}_{j}+{Treatment:Stage\;of\;lactation}_{ij}+{Cow(Treatment)}_{k}+\epsilon_{ijk}$$

where *Y_ijk_* was a dependent variable of cow *k* within treatment *i* at stage of lactation *j*. Treatment, stage of lactation and their interaction were fixed factors, and cow was a random factor. Measurements repeated over a stage of lactation were considered by using a covariance matrix. The choice of the structure of the matrix was determined according to the structure of the data and by using the Akaike information criterion for analyses of all variables. The average flows of Ca and P during the 4 days of measurements were also analyzed independently during each stage of lactation with a generalized linear model using PROC GLM in SAS and included only the fixed effect of treatment. These analyses supplemented the previous analyses performed with the complete model because it has been shown that with a smaller quantity of data, the inclusion of repeated measurements in a generalized linear mixed model can limit the detection of significant effects by increasing the type II error [36]. Data other than average flows of Ca and P were also analyzed independently with a generalized linear model during each of the 3 stages of the mineral balance measurements (-3 weeks, 3 weeks and 17 weeks of lactation) using PROC GLM in SAS, including only the fixed effect of treatment. As these analyses did not demonstrate a significant treatment effect, those results will not be presented.

## Results

**Fig 1:**
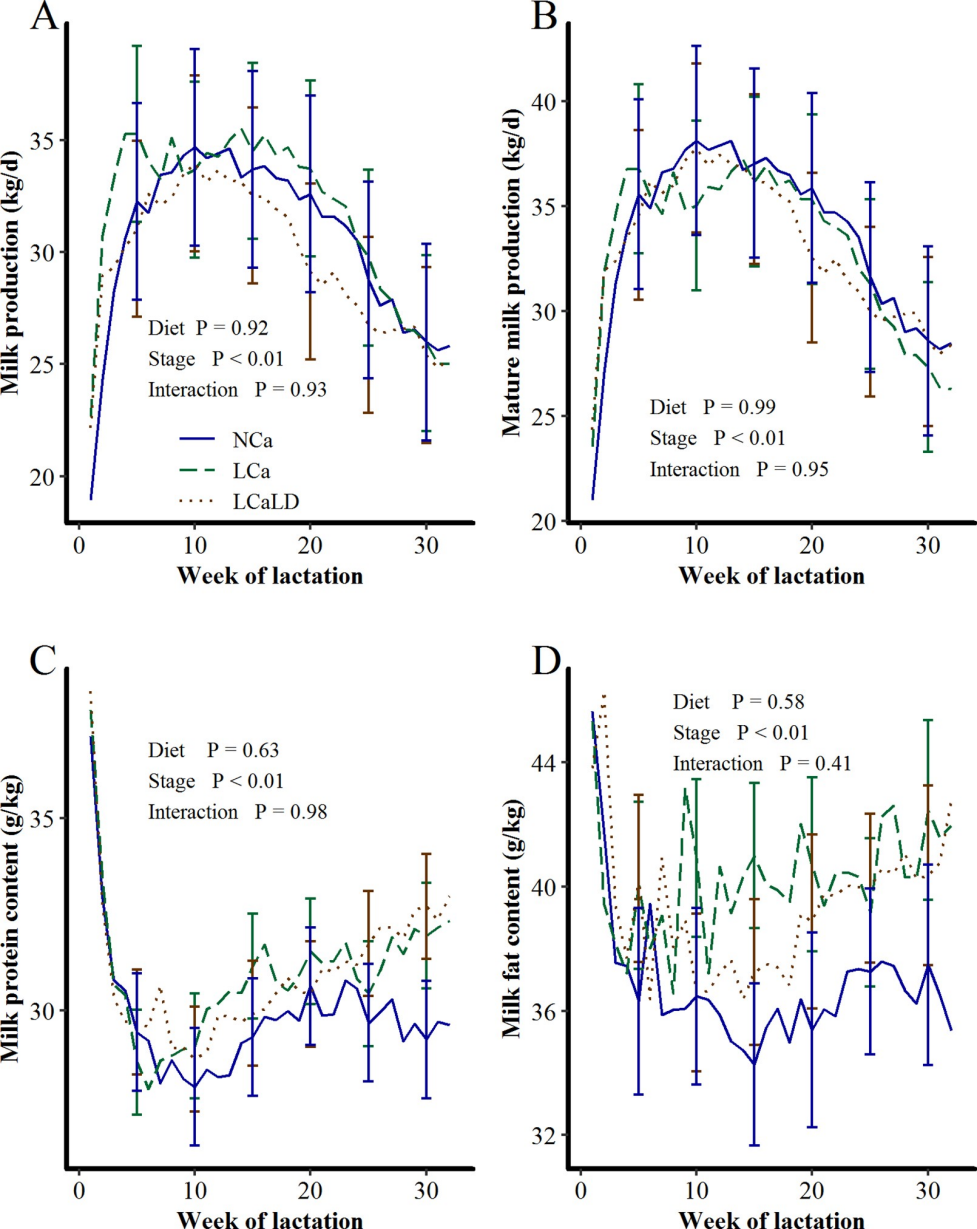
**Differences in A) milk production, B) mature equivalent milk production, C) milk protein content and D) milk fat content among cows during the first 32 weeks of the lactation preceding that described in the present paper according to their subsequent treatments.** NCa = normal Ca (4 cows), LCa = low Ca (5 cows), LCaLD = low Ca and low DCAD (5 cows). Data were analyzed using a generalized linear mixed model with repeated values using PROC GLIMMIX in SAS[35]. Covariance matrix: SP for milk production, mature milk production, and milk protein content and FA for milk fat content. Vertical bars represent standard errors of the mean. Standard errors are shown every 5 weeks for readability.

The distribution of the cows among the 3 treatments had to be modified before the differentiation of the TMR 5 days after calving. Two cows calved more than 2 weeks before the expected calving: one was assigned to the NCa treatment, and the other was assigned to the LCaLD treatment. These cows were removed from the experiment and replaced by the extra cows in the considered groups, and only one blood sampling was performed before calving for each replacement cow. Another cow, initially assigned to the LCaLD treatment and diagnosed with milk fever, was replaced by a cow initially assigned to the NCa treatment and belonging to the same calving date group. At the 7^th^ week of lactation, one cow in the NCa treatment died due to bowel obstruction, and its data were removed from the data set. Despite these events, the pre-experimental characteristics of the 3 experimental lots remained similar. The average parities were 2.4 for the LCaLD and 3.0 for the NCa and LCa. Milk production during the first 32 weeks of the previous lactation was not affected by the treatments (Fig 1A, P = 0.92 for the treatment effect and P = 0.93 for the treatment × stage of lactation interaction effect). Mature milk yield was not different between the treatments (Fig 1B, P = 0.99 for the treatment effect and P = 0.95 for the treatment × stage of lactation interaction effect). The average mature equivalent milk production over the first 32 weeks of lactation was 33.6 ± 3.94, 33.2 ± 3.52 and 33.1 ± 3.52 kg/d for the NCa, LCa and LCaLD treatments, respectively. Neither milk protein content (Fig 1C, P = 0.62) nor milk fat content (Fig 1D, P = 0.58) was affected by the treatments during the first 32 weeks of the previous lactation, but the variability in the last two parameters was high. The average milk protein contents over the first 32 weeks of lactation were 29.8 ± 0.97, 31.0 ± 0.87 and 31.0 ± 0.87 g/kg for the NCa, LCa and LCaLD treatments, respectively. The average milk fat contents over the first 32 weeks of lactation were 36.8 ± 2.48, 40.3 ± 2.22 and 39.3 ± 2.22 g/kg for the NCa, LCa and LCaLD treatments, respectively.

### Ca, P and DM intake

**Fig 2:**
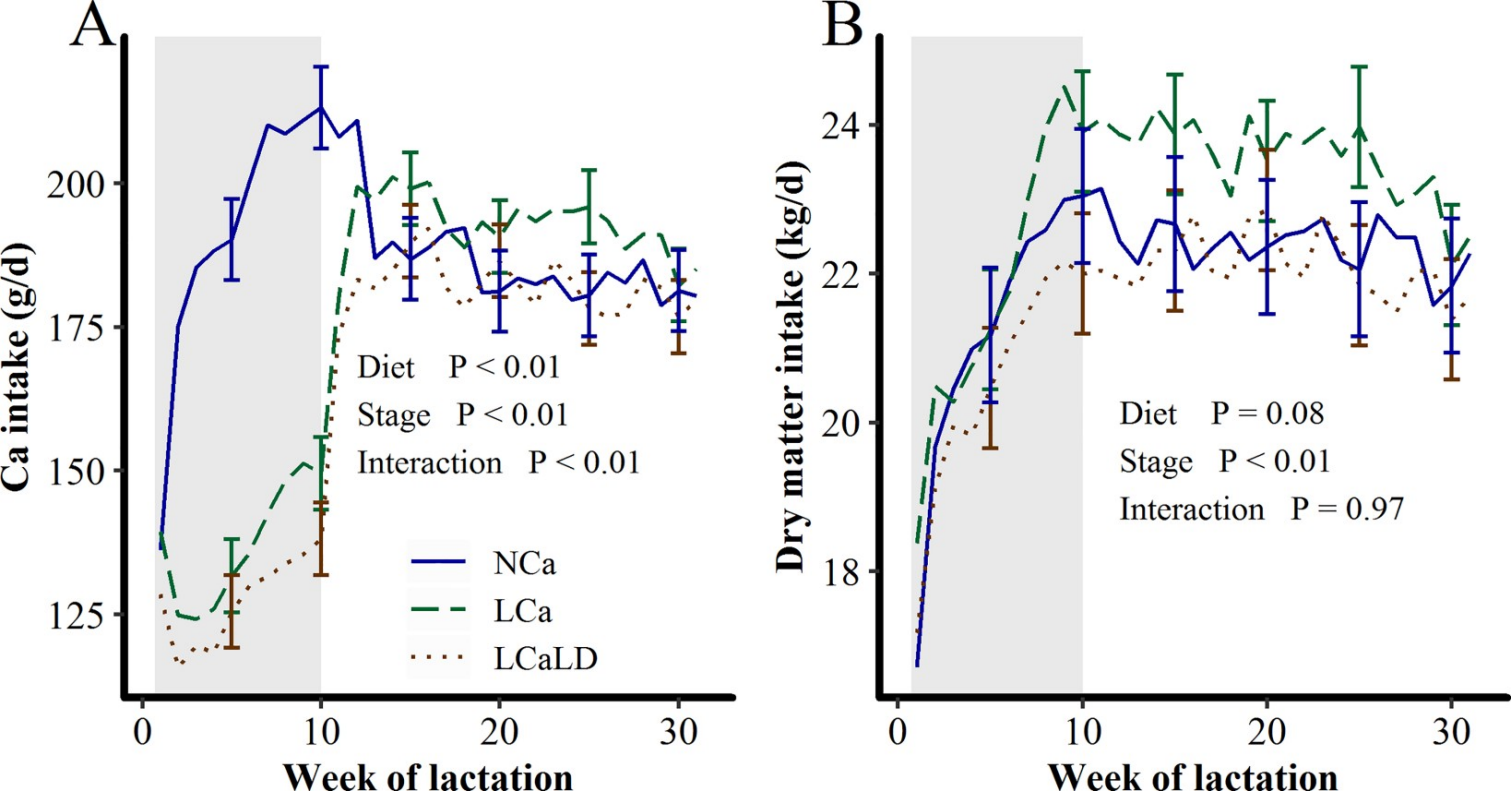
**Effect of transient variation in dietary Ca content and the DCAD on A) Ca intake and B) dry matter intake between calving and 32 weeks of lactation**. NCa = normal Ca (4 cows), LCa = low Ca (5 cows), LCaLD = low Ca and low DCAD (5 cows). Gray background specifies the period during which cows were fed differentiated diets according to the treatments. Data were analyzed using a generalized linear mixed model with repeated values using PROC GLIMMIX in SAS [35]. Covariance matrix: SP for Ca intake and dry matter intake. Vertical bars represent standard errors of the mean. Standard errors are shown every 5 weeks for readability.

During the period when the experimental diets were fed, from the 5^th^ to 70^th^ day of lactation, the Ca intake was significantly lower with the LCa and LCaLD treatments compared with the NCa treatment (P < 0.01). During this period, the average daily intake of Ca was 136.0 ± 4.98 g/d and 126.5 ± 4.98 g/d for LCa and LCaLD, respectively, and 184.1 ± 5.06 g/d for NCa, leading to a Ca intake that was 31% lower for the LCa and LCaLD treatments compared with the NCa treatment, as expected. During the same period, the difference between absorbable Ca intake and Ca requirements calculated according the INRA feeding system [15] was negative for the LCa and LCaLD treatments (-13.4 ± 1.36 and -15.5 ± 1.36 g/d, respectively), whereas it was positive for the NCa treatment (5.4 ± 1.42 g/d, P < 0.01, Fig 2A). After the 70^th^ day of lactation, when cows received the same diet, the Ca intake was not affected by the treatments, and the difference between absorbable Ca intake and Ca requirements always remained positive, with an average value of 10.9 ± 1.12 g/d (Fig 2A). The DM intake tended (P = 0.08, Fig 2B) to be higher for the LCa treatment compared with the LCaLD and NCa treatments, with DM intakes of 23.0 ± 0.4, 21.6 ± 0.4 and 22.0 ± 0.5 kg/d for the LCa, LCaLD and NCa treatments, respectively, throughout the lactation. Consequently, the P intake also tended (P = 0.08, see S1 Table) to be higher with the LCa treatment compared with the LCaLD and NCa treatments across lactation, with a P intake of 91.8 ± 1.70, 86.1 ± 1.7 and 89.4 ± 1.90 g/d for the LCa, LCaLD and NCa treatments, respectively. The amount of P ingested by the cows exceeded the requirement by 4.09 ± 0.90 g/d during the first 70 days of lactation.

### Plasma concentrations of biomarkers of bone formation and resorption, Ca and Pi

**Fig 3:**
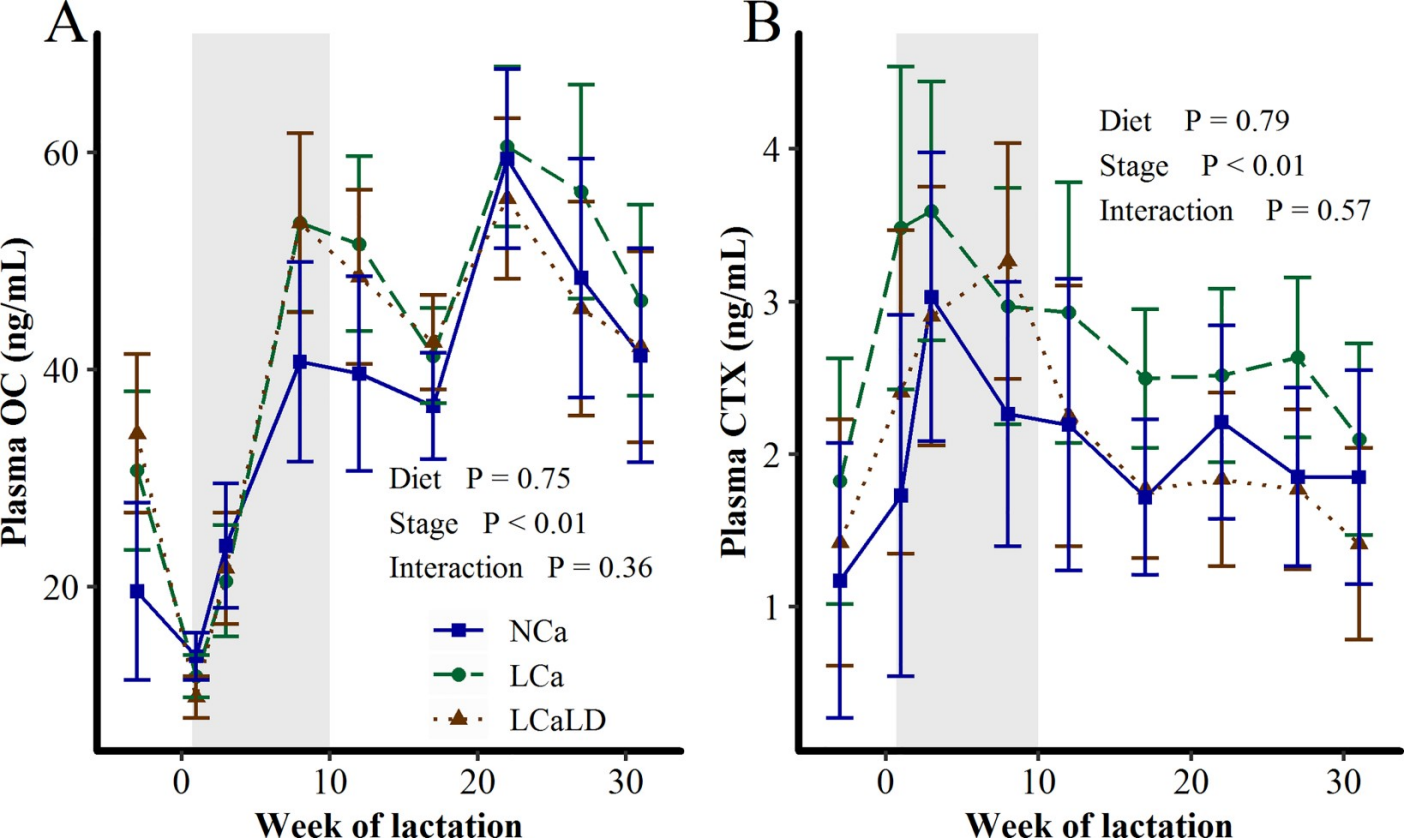
**Effect of transient variation in dietary Ca content and the DCAD on A) plasma OC concentration and B) plasma CTX concentration between 3 weeks before calving and 31 weeks of lactation.** NCa = normal Ca (4 cows), LCa = low Ca (5 cows), LCaLD = low Ca and low DCAD (5 cows). Gray background specifies the period during which cows were fed differentiated diets according to the treatments. Data were analyzed using a generalized linear mixed model with repeated values using PROC GLIMMIX in SAS [35]. Covariance matrix: FA for plasma OC content and UN for plasma CTX content. Vertical bars represent standard errors of the mean.

The plasma concentrations of OC and CTX were affected neither by the treatments nor by the treatment × stage of lactation interaction (Fig 3). For all treatments, plasma OC concentration decreased after calving to reach a minimal value one week after calving, whereupon it increased sharply for 10 weeks and then more slowly until the end of lactation (stage of lactation effect, P < 0.01). For all treatments, the plasma CTX concentrations increased sharply after calving to reach a maximal value between weeks 3 and 8 of lactation and then decreased until the end of lactation (stage of lactation effect, P < 0.01). The plasma Ca concentration was 100.3 ± 1.84 mg/L on average, and individual values always remained between 80 and 120 mg/L, with only one cow exhibiting hypocalcemia at one week of lactation (76 mg/L). The plasma Ca concentration tended to be lower at 1 and 3 weeks of lactation than at the other sampling times (stage of lactation effect, P = 0.06, see S1 Table) but was affected neither by the treatments (P = 0.63) nor by the treatment × stage of lactation interaction (P = 0.31). The plasma Pi concentration was 50.7 ± 1.78 mg/L on average. Some individual values were lower than 40 mg/L at 3 weeks of lactation, but no individual values were higher than 80 mg/L. Plasma Pi sharply decreased after calving at 1 week and 3 weeks of lactation, increasing afterward and leveling off at 17 weeks of lactation (stage of lactation effect, P < 0.01, see S1 Table). It was affected neither by the treatment (P = 0.89) nor by the treatment × stage of lactation interaction (P = 0.63).

### Ca and P partitioning and retention

**Fig 4:**
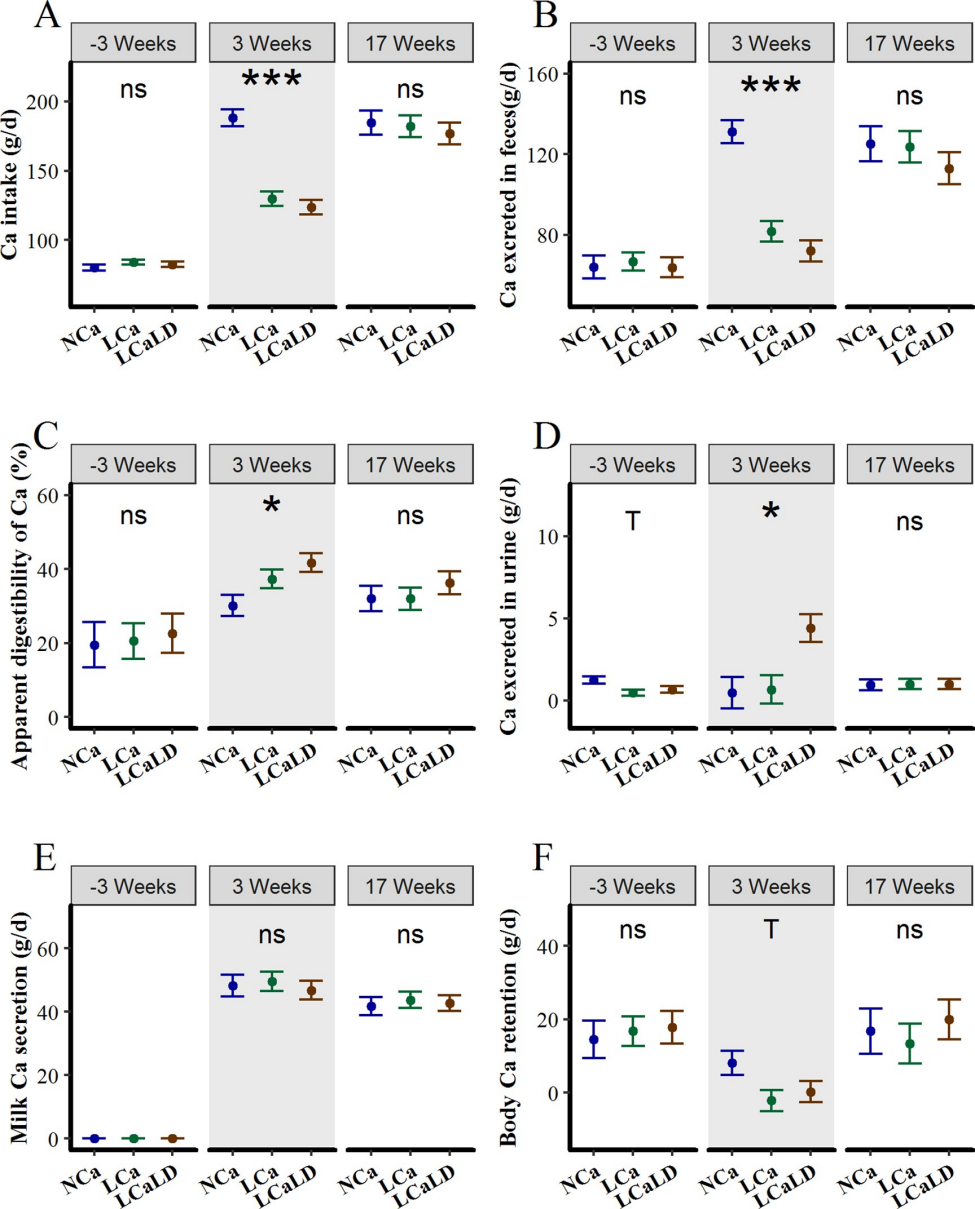
**Effect of transient variation in dietary Ca content and the DCAD on A) daily Ca intake, B) fecal losses of Ca, C) apparent digestibility of Ca, D) urinary losses of Ca, E) Ca secretion in milk and F) Ca balance at 3 weeks before and after calving and at 17 weeks after calving.** NCa = normal Ca (4 cows), LCa = low Ca (5 cows), LCaLD = low Ca and low DCAD (5 cows). Gray background specifies the period during which cows were fed differentiated diets according to the treatments. Data were analyzed according to a one-way ANOVA using PROC GLM in SAS [35] at each stage of lactation, considering only the fixed effect of treatment. Symbols represent P-values of the treatment effect: ns = not significant, i.e., P > 0.10; T = tendency, i.e., P < 0.10; * = significant, i.e., P < 0.05; ** = significant, i.e., P < 0.01; and *** = significant, i.e., P < 0.001. Vertical bars represent standard errors of the mean.

During the 4 days of measurement of Ca and P retention, the Ca intake was lower for the LCa and LCaLD treatments compared with the NCa treatment at 3 weeks of lactation, i.e., during the period of TMR differentiation between treatments (P < 0.05, Fig 4A), whereas the Ca intake was not affected by the treatment 3 weeks before calving or at 17 weeks of lactation (P > 0.10 for both stages, treatment × stage of lactation interaction: P < 0.001). The daily amount of Ca excreted in the feces was also lower for the LCa and LCaLD treatments compared with the NCa treatment at 3 weeks of lactation (P < 0.001, Fig 4B) and was not affected by treatment 3 weeks before calving or at 17 weeks of lactation (P > 0.10 for both stages, treatment × stage of lactation interaction: P < 0.001). The apparent digestibility of the Ca increased after calving (Fig 4C, P < 0.001), from 21.0% ± 2.33 3 weeks before calving to 36.4% ± 2.10 and 33.4% ± 2.10 at 3 and 17 weeks of lactation, respectively. The apparent digestibility of Ca was not affected by the treatments 3 weeks before calving or at 17 weeks of lactation, but it was higher for the LCa and LCaLD treatments compared with the NCa treatment at 3 weeks of lactation (P = 0.03, average of 37.3 ± 3.5% for LCa, 41.7 ± 3.5% for LCaLD and 30.1 ± 3.9% for NCa). The daily amounts of Ca excreted in urine were low, specifically 2.00 g/d on average, compared to the other flows of the input-output retention, as expected, and this flow tended to be affected by only the stage of lactation (P = 0.09, Fig 4D). However, the daily amount of Ca excreted in the urine was higher with the LCaLD treatment compared with the NCa treatment and the LCa treatment at 3 weeks of lactation (P < 0.05, treatment × stage of lactation interaction: P = 0.004, 4.4 ± 0.5 g/d for LCaLD vs. 0.5 ± 0.6 g/d for NCa and 0.7 ± 0.5 g/d for LCa). The daily amount of Ca secreted in milk was on average 48.2 ± 1.42 g/d at 3 weeks of lactation and 42.7 ± 1.42 g/d at 17 weeks (Fig 4E). The amount of Ca secreted in milk daily decreased slightly between 3 and 17 weeks of lactation (P < 0.001) and was not affected by the treatments at any stage. Daily Ca retention, i.e., the difference between the input and output of Ca, was strongly positive before calving (+17.3 ± 2.84 g/d, Fig 4F), decreased at 3 weeks of lactation to an average value close to 0 (2.1 ± 2.62 g/d) and increased at 17 weeks of lactation to a positive value (+16.7 ± 2.62 g/d, stage of lactation: P < 0.001). At 3 weeks of lactation, the daily Ca retention tended to be lower for the LCa and LCaLD treatments compared with the NCa treatment (P = 0.09), with values around equilibrium for the LCa and LCaLD (-2.1 ± 4.3 g/d for LCa, + 0.3 ± 4.3 g/d for LCaLD and + 8.1 ± 4.8 g/d for NCa). The daily Ca retention was not different among the 3 groups of cows assigned to the 3 treatments 3 weeks before calving or at 17 weeks of lactation (P > 0.10).

DM intake, P intake, and fecal excretion of DM and P were not affected by treatment or the treatment × stage of lactation interaction (P > 0.10, see Table 1). The apparent digestibility of both the DM and P increased at calving and leveled off after 3 weeks of lactation. The apparent DM digestibility was 65.1 ± 0.86 at 3 weeks before calving, 72.7 ± 0.79% at 3 weeks of lactation and 71.6 ± 0.79% at 17 weeks of lactation (P < 0.001), and the apparent P digestibility was 10.9 ± 2.17% 3 weeks before calving, 54.7 ± 1.98% at 3 weeks of lactation and 47.5 ± 1.98% at 17 weeks of lactation (P < 0.001). Daily P retention tended to increase at 17 weeks of lactation compared with the two other stages of lactation, with values of 4.3 ± 1.66 g/d at 3 weeks before calving, 7.9 ± 1.48 g/d at 3 weeks of lactation and 10.0 ± 1.48 g/d at 17 weeks of lactation (P = 0.08).

### Milk production and composition

**Fig 5:**
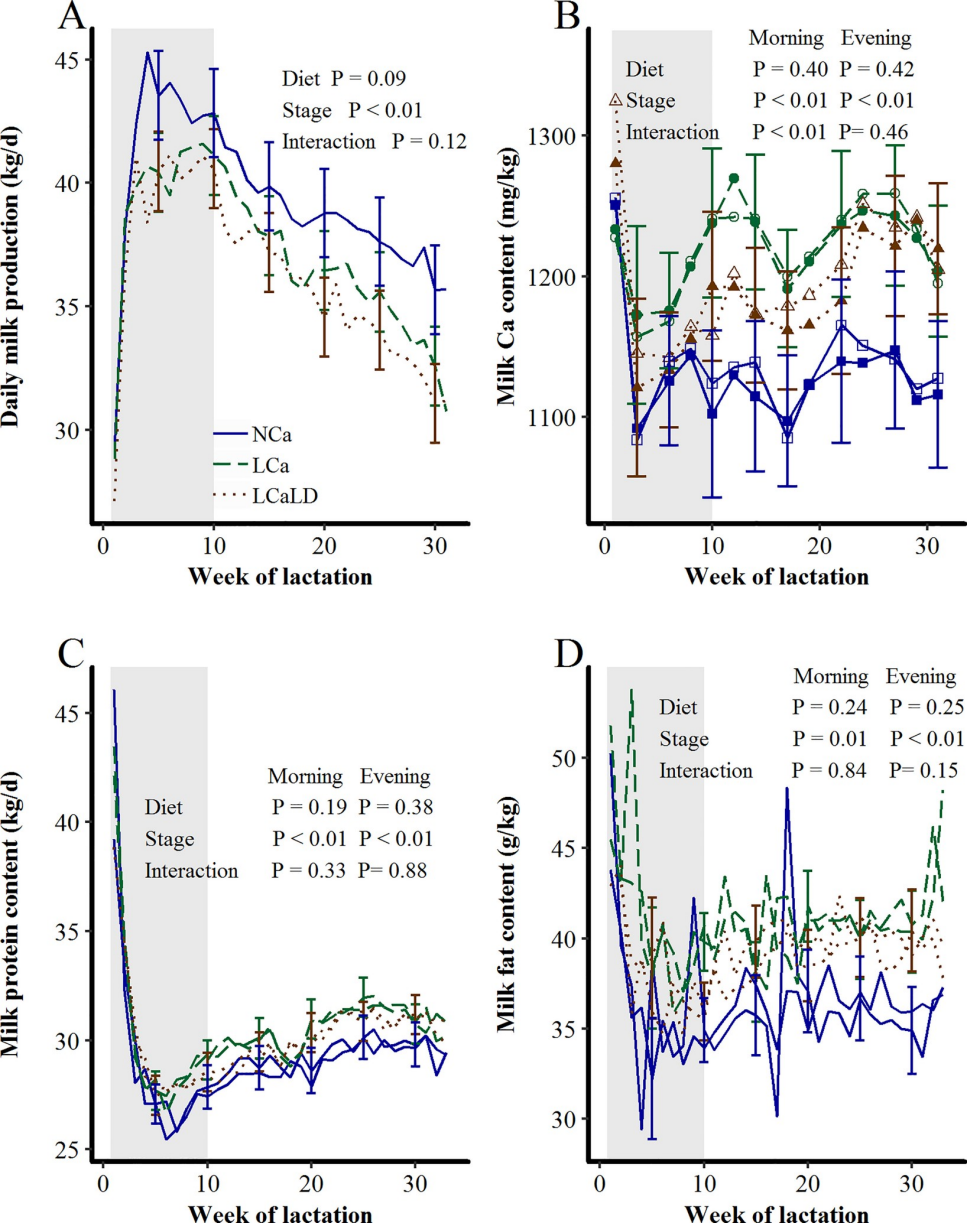
**Effect of transient variation in dietary Ca content and the DCAD on A) daily milk production, B) milk Ca content, C) milk protein content and D) milk fat content between calving and 32 weeks of lactation.** For figure B, color-filled shapes = morning milk Ca content, and white-filled shapes = evening milk Ca content. Treatments: NCa = normal Ca (4 cows), LCa = low Ca (5 cows), LCaLD = low Ca and low DCAD (5 cows). Gray background specifies the period during which cows were fed differentiated diets according to the treatments. Data were analyzed using a generalized linear mixed model with repeated values using PROC GLIMMIX in SAS [35]. Covariance matrix: SP for milk production and morning milk protein content and FA for morning and evening milk Ca contents, evening milk protein content and morning and evening milk fat contents. Vertical bars represent standard errors of the mean. Standard errors are shown every 5 weeks and in the morning for milk production, milk protein and fat contents for readability. The standard error for milk Ca content is shown every 2 weeks in the morning for readability.

Milk production tended to be lower throughout the 32 weeks of lactation for the LCa and LCaLD treatments compared with the NCa treatment (P = 0.09, Fig 5A), with average values of 36.8 ± 0.9 kg/d for LCa, 35.9 ± 0.9 kg/d for LCaLD and 39.2 ± 1.1 kg/d for NCa. This production led to a difference in cumulative milk production at 200 days of lactation between the low-Ca treatments (LCa and LCaLD) and the control treatment (NCa) of more than 400 kg. The difference in milk production between the low-Ca treatments and the NCa treatment was maximal at the 4^th^ week of lactation, with a milk production difference of more than 4.5 kg/d. The milk Ca content sharply decreased to reach a minimal value at 3 weeks of lactation (stage of lactation effect, P < 0.01, Fig 5B). After 8 weeks of lactation, the milk Ca content increased for the LCa and LCaLD treatments, whereas it remained stable for the NCa treatment (stage of lactation × treatment interaction: P < 0.01 in the morning and nonsignificant in the evening). After the 24^th^ week of lactation, the morning milk Ca contents were 1230 ± 48.4 and 1228 ± 48.4 mg/kg for the LCa and LCaLD treatments, respectively, and 1128 ± 54.2 mg/kg for the NCa treatment (P < 0.01). The milk protein content was numerically higher for cows fed the LCa and LCaLD treatments compared with those fed the NCa treatment between 5 and 9 weeks of lactation and after 20 weeks of lactation, but the treatment effect was not significant (Fig 5C). Milk fat content was also unaffected by the stage of lactation × treatment interaction (Fig 5D, P > 0.10).

### Milk Ca and protein partitioning between soluble and colloidal phases

**Fig 6:**
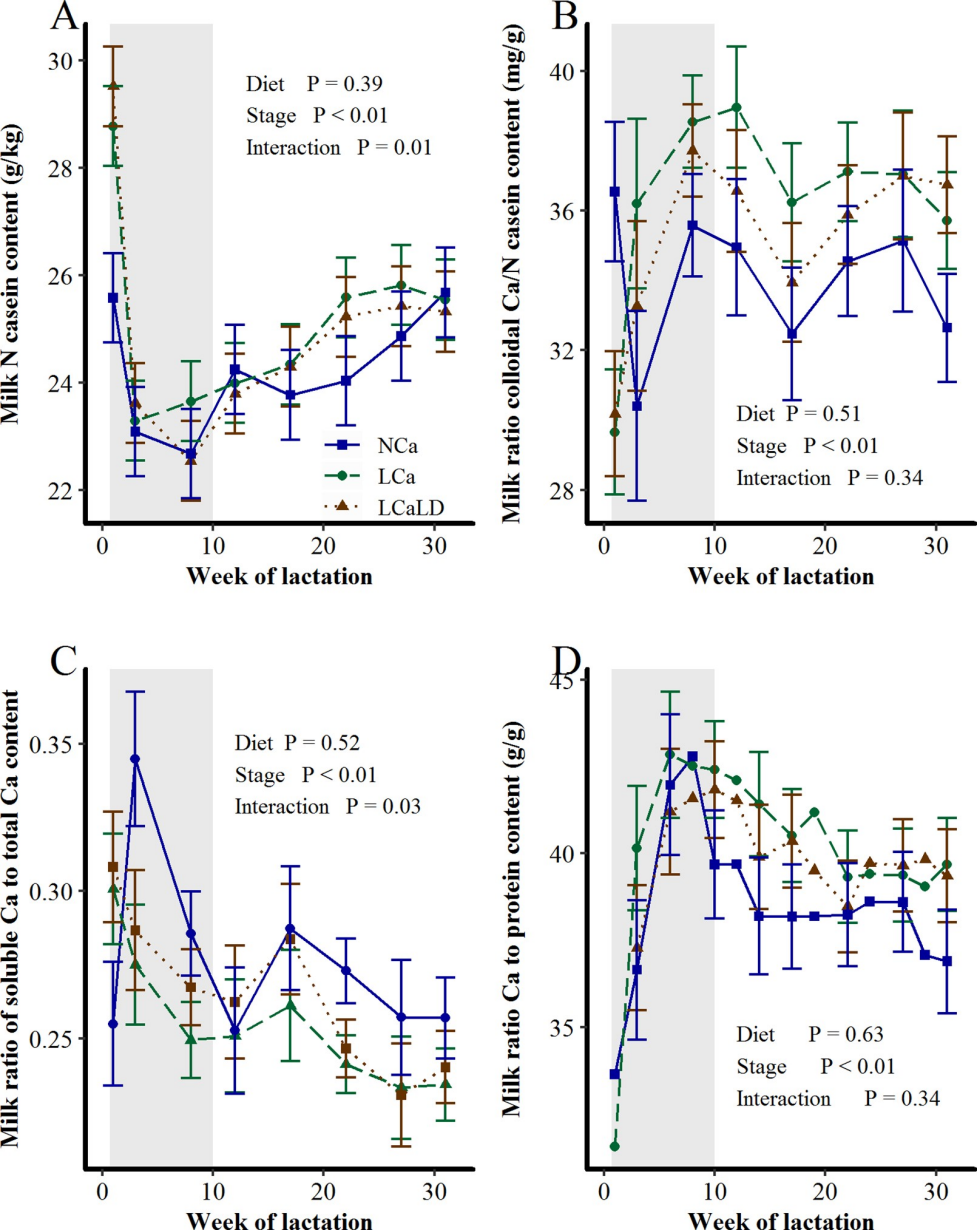
**Effect of transient variation in dietary Ca content and the DCAD on the morning milk A) casein content, B) ratio of colloidal Ca to casein content, C) ratio of soluble Ca to total Ca content and D) ratio of Ca to protein content between calving and 32 weeks of lactation.** NCa = normal Ca (4 cows), LCa = low Ca (5 cows), LCaLD = low Ca and low DCAD (5 cows). Gray background specifies the period during which cows were fed differentiated diets according to the treatments. Data were analyzed using a generalized linear mixed model with repeated values using PROC GLIMMIX in SAS [35]. Covariance matrix: SP for milk casein content, un for milk ratio of colloidal Ca to casein content and FA for ratios of soluble to total Ca content and Ca to protein content. Vertical bars represent standard errors of the mean. Standard errors are shown for every two measurements for the milk Ca to protein content ratio for readability.

The milk casein content was higher with the LCa and LCaLD treatments compared with the NCa treatment after 17 weeks of lactation (Fig 6A, stage of lactation × treatment interaction, P < 0.01). At the end of the period of diet differentiation between treatments, the milk casein content of the NCa treatment increased transitorily. The ratio between the milk contents of colloidal Ca and casein increased at the beginning of lactation and remained relatively stable after 8 weeks of lactation, at a value approaching 36 mg/g (Fig 6B). This ratio was not affected by the treatments or the stage of lactation × treatment interaction (P > 0.10). The proportion of soluble Ca was lower for the LCa and LCaLD treatments compared with the NCa treatment during early lactation (Fig 6C, interaction: P < 0.01). This proportion decreased transitorily at the end of the period of diet differentiation between treatments for the NCa treatment (stage of lactation × treatment interaction, P = 0.03). On average, 27% of the milk Ca was in the soluble form. The ratio between the milk Ca and protein contents increased at the beginning of lactation to peak at approximately 6–8 weeks of lactation and then decreased to level off after 17 weeks of lactation to a value close to 39 mg/g on average (Fig 6D, P < 0.001). This value was affected neither by the treatment nor by the stage of lactation × treatment interaction.

## Discussion

### Limited effect of dietary Ca supply and DCAD on the dynamics of bone mobilization and reconstitution during lactation

Our results describe a dynamic process of bone mobilization throughout lactation, which is consistent with the literature. The dynamics of the blood-bone biomarkers throughout lactation consisted of a sharp decrease in OC at calving and an increase in CTX at the beginning of lactation, as in other studies [10,11,13,25]. Because OC is a biomarker of bone accretion and CTX is a biomarker of bone resorption, these results are indicative of relative bone mobilization and the beginning of lactation and relative bone reconstitution at the end of the measurement period. This finding was confirmed by the lower Ca retention that we observed at the beginning of lactation compared to that measured before calving or at 17 weeks of lactation.

Another expected result would have been that the LCa and LCaLD treatments induced increased bone mobilization during the first 10 weeks of lactation. However, only a small decrease in Ca retention was observed 3 weeks after calving with those treatments compared to the control treatment (NCa), with no effect on the dynamics of the blood biomarkers of formation and resorption throughout lactation. Even at 3 weeks of lactation, the Ca retention remained close to 0 in the LCa and LCaLD treatments and was not clearly negative. For the LCa and LCaLD treatments, the dietary Ca supply was limited to 70% of the recommended supply needed to fulfill the cows’ requirements according to the expected milk production and intake according to the INRA feeding system [15]. Other studies highlighted an increase in bone mobilization with such Ca supply restrictions compared to a control, which could be deduced from either a decrease in the body retention of Ca at the beginning of lactation in dairy cows to negative values [11] or an increase in the serum concentration of pyridinoline, a biomarker of bone resorption [19]. The DCAD of the LCaLD treatment approached 0 mEq / kg DM, which is within the recommended values for a sufficient effect on bone mobilization for the prevention of milk fever without affecting intake in late-gestating cows [21,37,38]. Although the recommended range of the DCAD for the prevention of milk fever used to be between -50 and -150 mEq/kg DM [21], it was subsequently suggested that it was not beneficial to reduce DCAD below 0 mEq/kg due to a negative effect on intake [37,38]. In our experiment, the LCaLD treatment was applied to cows at the very beginning of lactation, and we chose not to risk an additional limit to ingestion. The expected effect of lowering the DCAD was a decrease in the pH of the blood, to avoid changes in PTH receptor conformation, to preserve the receptor affinity for PTH in tissue [39] and to favor the release of Ca from bone carbonates, which constitute a buffering system when the local pH is low [40]. In Jersey cows, high-DCAD diets reduce tissue sensitivity to PTH compared to low-DCAD diets and thus diminish calcium homeostatic responses [39]. In our experiment, only slight calcium homeostatic responses were observed in bones with the LCa treatment. The slight calcium homeostatic responses in bones also observed with the LCaLD treatment could mean that this leverage did not have to be involved in calcemic regulation in our experiment and thus could not be increased by increased bone tissue sensitivity to PTH. Finally, it is also interesting to note that the diet offered to the cows during the first 10 weeks of lactation in our experiment was intentionally formulated with a high dietary PDI to NE_L_ ratio to maximize the milk production capacity of the mammary gland, which should have increased the necessity of bone mobilization [25].

### Dairy cows have adapted to low dietary Ca supplies by increasing digestive absorption of Ca during early lactation

In our experiment, as in those of Taylor et al. [11] and Braithwaite [18], the evolution of calcemia during the lactation-gestation cycle was not affected by the dietary Ca supply, which confirms that calcemia is tightly regulated. This finding suggests that if bone mobilization was not the main effector mobilized for the regulation of calcemia when the Ca supply was lowered in our experiment, other Ca flows allowed this regulation. Our results clearly illustrate that the decrease in Ca intake with the LCa and LCaLD treatments was almost entirely compensated for at the organismal scale by an equivalent decrease in the daily amount of Ca excreted in feces, i.e., by increased apparent digestive absorption of Ca. The apparent digestibility of Ca was notably high, with an average greater than 40% for the LCaLD treatment. These results contrasted with those of Taylor et al. [11] and Moreira et al. [19], who observed lower apparent digestibility of Ca at a similar stage of lactation, with the highest values being approximately 35%. A reason for this finding may be that a significant proportion of the dietary Ca was provided by alfalfa silage or hay in those studies, whereas it was mainly provided by a mineral source of Ca in ours. Ca from alfalfa is known to be less available for absorption in ruminants [5]. It is possible that the reason that Taylor et al. [11] and Moreira et al. [19] observed an increase in bone mobilization at the very beginning of lactation under conditions of a low calcium supply was because the cows could not increase their apparent absorption of Ca from a diet with limited availability of dietary Ca. In our experiment, calcemia regulation may have been possible due to an increase in digestive absorption without increased mobilization of Ca from bone, likely because dietary Ca was more available for absorption. This hypothesis warrants further investigation. A clear increase in digestive absorption of Ca at the beginning of lactation with a low dietary supply of Ca has also been observed in ewes [18], but in that study, a concomitant increase in bone mobilization was also observed. This finding can be explained by the important restriction of the Ca supply compared to that in the aforementioned experiments [11,19] or in ours, which likely necessitated the implementation of several adaptive mechanisms.

We observed a clear, strong effect of cow physiological stage on the apparent digestibility of Ca, with higher digestibility at 3 or 17 weeks of lactation compared with 3 weeks before calving. The increase in Ca absorption capacity of the digestive tract between gestation and lactation agrees with the increase in PTH release and 1,25-(OH)_2_D_3_ synthesis at the onset of lactation [2] but has not been clearly illustrated previously. We also observed a clear positive effect of the LCaLD treatment on the daily amount of Ca excreted in urine, but the size of the flow, i.e., less than 4 g/d at 3 weeks of lactation, was limited compared with that of Ca excreted in milk (approximately 50 g/d) or in feces (approximately 100 g/d). The increase in this flow with the treatment providing a DCAD close to 0 agrees with the idea that a high hydrogen ion content in the glomerular filtrate interferes with the ability of the kidneys to reabsorb Ca from the filtrate, causing an increase in urinary Ca excretion [39].

### Relationship between the dynamics of milk Ca content and bone formation and resorption during lactation

Experiments with lactating mice have demonstrated that a decrease in Ca intake can induce an increase in bone resorption and a concomitant decrease in the milk Ca to protein ratio [27]. These effects have been shown to be mediated by the CaSR of the epithelial cells of the mammary gland, with a lack of Ca on the CaSR decreasing Ca transport into the milk and increasing PTHrP secretion by the mammary gland and thus bone resorption. Our first hypothesis was that low Ca intake would induce both an increase in bone resorption and a decrease in the secretion of Ca in milk. However, the effects of the low Ca supply on bone mobilization at the beginning of lactation in our experiment were limited; thus, this experiment did not enable thorough testing of our second hypothesis, which was that a decrease in the milk Ca content would be concomitant with bone mobilization.

The cows affected by the LCa and LCaLD treatments tended to have a higher milk Ca content after 10 weeks than those in the control treatment, which did not agree with our hypothesis for 2 reasons. First, this difference appeared when the diet was similar for all treatments. A lower milk Ca content was expected for those treatments. Because genetics is known to be a major determinant of milk Ca content in cows [41] and because the milk Ca content was not measured prior to the previous lactation, when cows were assigned to the treatments, the cows in the NCa treatments possibly had lower milk Ca contents because of their genetics. The milk casein contents of these cows were also lower during late lactation, and most milk Ca was bound to casein [42], with a stable ratio between colloidal Ca and casein, as was observed in our experiment. However, we also observed a highly transient change in the proportion of the milk-soluble Ca and even of the total milk Ca contents when the TMR changed at 10 weeks of lactation. This finding suggests that a decrease in milk Ca content may be an initial transient response to a decrease in Ca flow to the blood. Thus, the milk Ca content may not be a relevant indicator of the whole pattern of bone resorption at the scale of lactation, but its decrease may be a transient indicator of Ca deficiency. This finding cannot be established from our experiment because of the sampling interval of 2 weeks, but it warrants further research.

### Possible effect of restricted Ca intake on milk production and cow longevity

The difference of 2 kg/d in milk production between the NCa treatment in one direction and the LCa and LCaLD treatments in the other direction was an unexpected result that could not be attributed to the pre-experimental measurements of the cows. The discrepancy between the treatments appeared approximately 2 weeks after the differentiation of the diets according to the treatment, i.e., at 3 weeks of lactation, and lasted until the end of the experiment, i.e., largely after 10 weeks of lactation, when all cows began to receive the same diet. It is impossible to draw firm conclusions regarding the effect of the dietary Ca supply on milk production from our experiment, given the small number of cows involved. However, whether the low Ca intake impaired potential milk secretion by the mammary gland at peak lactation by altering either the proliferation of the mammary epithelial cells or their exfoliation merits further investigation. Ca has been shown to function as an important messenger in cell proliferation, notably for the breakdown of the nuclear envelope [43]. It is possible that the milk production potential of cows in the LCa and LCaLD treatments was not totally expressed because Ca limited the capacity for cell proliferation during early lactation. Wohlt et al. [44] also observed a decrease in the milk production of cows with a lower Ca supply, with dietary Ca contents between 0.9 and 0.6% DM, but the response depended on the Ca source. Taylor et al. [11] did not observe any effect of dietary Ca content on milk production, but two-thirds of the cows were primiparous. Moreira et al. [19] did not observe such an effect with multiparous cows, but their experiment stopped after one month of lactation. Older studies [45] highlighted an effect of Ca supplementation on the milk production of dairy cows, but bone meal was used for Ca supplementation and also contained P, which is known to affect DM intake and milk production. Possibly because we used a high level of protein supplementation, with formaldehyde-treated soybean meal being partially protected from protein ruminal degradation, and multiparous cows, the milk production potential may have been maximized, and the Ca could have been a limiting factor. However, this possibility remains to be confirmed with more animals.

Another unexpected observation in our experiment was that the culling rate of cows before the next calving was clearly numerically higher in the LCa and LCaLD treatments than in the NCa treatment. In the LCa treatment, 3 of the 5 cows were culled before the next lactation, 1 because of the absence of estrus detection and 2 because of claw disorders. In the LCaLD treatment, 1 of the 5 cows was culled because of artificial insemination failure. All cows in the NCa treatment were kept for subsequent lactation without health or reproductive problems before the calving. Due to the small number of cows involved in this experiment, affirmation of an effect of the dietary Ca content on cow reproduction and health from our results was not possible. Subclinical hypocalcemia during the first three days of lactation has a negative effect on the reproductive performance of cows. Our results suggest that a Ca supply lower than the required supply during the first weeks of lactation could also have a detrimental effect [46], but this possibility remains to be demonstrated with more animals.

## Conclusion

Lowering the dietary Ca content to between 0.8 and 0.6 g/kg DM clearly increased the apparent digestive absorption of Ca of the cows at 3 weeks of lactation but marginally affected the body retention of Ca, which remained nearly zero. This result suggests that bone mobilization in cows at the beginning of lactation can be unaffected by the supply of Ca, as long as the source of Ca is available for absorption and the supply is not lower than 70% of the Ca requirement. The low supply of Ca did not clearly affect the milk Ca content. However, the possibility remains that the low Ca level did not lower the milk production level and did not affect the reproductive performances of the cows and their probability of undergoing a subsequent lactation. These results need to be confirmed using more animals and suggest that Ca supplementation must be carefully checked at the beginning of lactation.

## Supporting information

S1 Table Estimated adjusted means (± Standard errors of the means) of all studied variables according to the treatment and the week of lactation. (XLSX)
